# A comparative study regarding antibiotic consumption and knowledge of antimicrobial resistance among pharmacy students in Australia and Sri Lanka

**DOI:** 10.1371/journal.pone.0213520

**Published:** 2019-03-13

**Authors:** M. H. F. Sakeena, Alexandra A. Bennett, Stephen J. Carter, Andrew J. McLachlan

**Affiliations:** 1 Sydney Pharmacy School, The University of Sydney, Sydney, New South Wales, Australia; 2 Department of Pharmacy, Faculty of Allied Health Sciences, University of Peradeniya, Peradeniya, Sri Lanka; 3 NSW Therapeutic Advisory Group (TAG), Sydney, New South Wales, Australia; University of New South Wales, AUSTRALIA

## Abstract

**Introduction:**

Antimicrobial resistance (AMR) is a major global health challenge. Pharmacists play a key role in the health care setting to support the quality use of medicines. The education and training of pharmacy students have the potential to impact on patterns of antibiotic use in community and hospital settings. The aim of this study was to investigate and compare antibiotic use and knowledge of antibiotics and AMR among undergraduate pharmacy students in Australian and Sri Lankan universities.

**Methods:**

A cross-sectional survey was conducted in Australian and Sri Lankan universities that offer a pharmacy degree. A paper-based survey was utilised in Sri Lanka and an identical survey distributed online among pharmacy students in Australia. Descriptive and comparative data analyses were performed.

**Results:**

476 pharmacy students from 14 universities in Australia and 466 students from 6 universities in SL completed the survey. Participants commonly reported previous antibiotic use [Australia (88%) and Sri Lanka (86%)]. The majority of students [Australia (89%) and Sri Lanka (77%)] reported they obtained antibiotics with a prescription. Australian pharmacy students correctly reported regarding optimal antibiotic use for certain disease conditions when compared to Sri Lankan students (*P*<0.05). A greater antibiotic knowledge level regarding AMR was found among Australian students compared to Sri Lankan students (p<0.05).

**Conclusion:**

This study provides an understanding about antibiotic consumption and knowledge on AMR among pharmacy students in a developed country, Australia and a developing country, Sri Lanka. These findings identify possible misconceptions about antibiotics and a lower level of knowledge of AMR amongst Sri Lankan undergraduate pharmacy students. Future research should focus on implementation of a strategic education plan for undergraduate pharmacy students in Sri Lankan universities. The curricula of pharmacy courses in Australian universities may inform such a plan.

## Introduction

Antimicrobial resistance (AMR) has been called the silent tsunami [1]. It will affect all countries, cost vast amounts of money, prolong illness and affect enormous numbers of lives [1]. In September 2015, World Health Organization (WHO) developed a United-Nations-endorsed Global Action Plan outlining the globally-approved national policy changes that are required to fight its potential devastating effects on the world’s population [2]. WHO has also identified the reduced capacity and lack of preparedness by many lower income countries to fight AMR and their need for greater support. A key strategic objective of the global action plan is to improve awareness and understanding of antimicrobial resistance through effective communication, education and training. The objective emphasises the need to make AMR a core component of professional education, training, certification, continuing education and development in health sectors. Pharmacists are medicine experts, trained to ensure quality use of medicines, including antimicrobials, and important members of the healthcare team [3]. There is potential for pharmacists to have greater impact mitigating AMR in the community and hospital settings with enhanced education and training [4]. Augmentation of current education and training of pharmacy students on the use of antibiotics and AMR is likely to be an efficient, effective and cost-effective strategy to influence appropriate behaviours by healthcare professionals and consumers regarding antibiotic use.

Australia and Sri Lanka offer a four-year undergraduate pharmacy degree (Bachelor of Pharmacy) and some Australian universities have a graduate entry Master of Pharmacy degree[5] [6]. In Australia, the role of pharmacists is well recognised and is considered an integral part of the health care system [7]. Furthermore, the pharmacists’ role continues to expand to provide a variety of services in both hospitals and community settings that includes: optimal medication use [8], which include antibiotic [9]; medication reconciliation [10]; disease state management [11]; comprehensive medication review [12] and, hospital discharge management and counselling [13]. Additionally, Australian undergraduate pharmacy curricula emphasize clinical pharmacy knowledge and skills and their application in the healthcare system [5]. On the other hand, in Sri Lanka, the role of pharmacists is still limited to traditional pharmacy activities such as procurement of drugs, extemporaneous compounding, dispensing of prescription medicines and selling of medicines. There is increasing recognition that Sri Lanka would benefit from delivery of expanded pharmaceutical care services and clinical pharmacy practice [14]. Expansion of the pharmacist’s role in the healthcare system is becoming more acceptable by healthcare professionals [15], [16]. Unlike Australia, Sri Lankan undergraduate pharmacy programs were introduced relatively recently [6], [17]. Teaching clinical pharmacy and practiced based modules in pharmacy curricula is challenging due to the lack of locally trained academic experts, limited hospital access for training and a paucity of clinical pharmacy services in the healthcare system [17].

The current study builds on our previous study that investigated knowledge of antibiotics and AMR among Sri Lankan pharmacy students [18]. This current study compares antibiotic usage and knowledge regarding antibiotics and AMR among undergraduate pharmacy students in Australia, a developed country and Sri Lanka, a developing country. The results of this study will be useful to identify gaps in pharmacy education and to inform interventions to improve pharmacists’ education and training on antibiotics and AMR. Therefore, the aim of this study is to compare self-reported use of antibiotics and knowledge of antibiotics and AMR in undergraduate pharmacy students of Australian and Sri Lankan universities.

## Materials and methods

### Study design and setting

A cross-sectional survey was conducted in Australian and Sri Lankan universities that offer a pharmacy degree. Pharmacy students from 17 Australian and 6 Sri Lankan pharmacy university courses were invited to participate in this study. A more detailed analysis of the data for Sri Lankan pharmacy students has been previously presented [18]. A paper-based survey was utilised between January and April 2016 in Sri Lanka. An identical online survey was distributed to pharmacy students from August to December 2017 in Australia. All male and female students enrolled in Bachelor of Pharmacy courses at the universities of Australia and Sri Lanka during the study period were eligible. Masters of Pharmacy students from Australian universities who took part in the survey were excluded from the current investigation. Any non-pharmacy undergraduate students participated inadvertently were also excluded from the analysis.

### Study size

This study aimed to achieve a representative sample of pharmacy students enrolled in pharmacy degree programs in each participated university in Australia and Sri Lanka. Convenient sampling technique was used to collect data. Total numbers of undergraduate pharmacy students enrolled during the data collection period in the fourteen Australian universities and six Sri Lankan universities were 5719 and 738 students, respectively. In this study we aimed for a minimum of 300 student responses from each country to allow a rigorous comparison between both countries.

### Data gathering

For the Sri Lankan phase study, a self–administered paper-based questionnaire was distributed to students following a short introduction to the research project and written informed consent. More details of the data collection procedure for Sri Lankan pharmacy students is given in our previously published paper [18]. For the Australian phase study, an e-mail invitation was sent to the Dean / Head or Coordinator of the pharmacy degree program at each university in Australia. An online survey questionnaire was used for the data collection (REDCap Software). An invitation to participate was distributed by e-mail to pharmacy students in their university. The email contained a link to the online survey and a participant information sheet. Reminder e-mails were sent twice to the university contact person, after a two or four weeks’ interval to increase the response rate. The study investigators did not have direct contact with the participants of the online survey.

### Data collection tool

The questionnaire used in this study is based on the World Health Organization (WHO): Antibiotic Resistance, Multi-country public awareness survey [19]. This questionnaire was selected because of its previous use by WHO and its comprehensive nature that included relevant topics of antibiotics use, knowledge on antibiotics and knowledge on antimicrobial resistance. Permission was obtained from WHO to reprint and reproduce the survey (WHO reference number 239656). The questionnaire was presented in English in both countries. The face validity of the online questionnaire was tested for readability, length and relevance of the questions amongst two pharmacists with postgraduate qualifications at Sydney Pharmacy School. Participants were presented with close ended questions and the questionnaire consisted with five major sections as following; basic demographic information: age, gender, name of the university and year of university study, antibiotic use: 4 questions about the use of antibiotics in the past, information on obtaining antibiotics, advice received at the time of purchase and the place, knowledge of antibiotics: 4 questions about the duration of treatment, knowledge on sharing antibiotics, symptoms justifying use of antibiotics and appropriate use of antibiotics in different disease conditions, knowledge of antibiotic resistance: 5 questions commonly used terms related to AMR and 8 True/False statements regarding knowledge of AMR and antibiotics use in the community: one question on antibiotic use in agriculture and food products.

### Ethical approval

The study was approved by the human ethics review committee, the University of Sydney, NSW, Australia (approval number 2016/868 dated: 13th December 2016) and by the institutional ethical clearance committee, Faculty of Medicine, University of Peradeniya, Peradeniya, Sri Lanka (Reference number 2015/EC/82 dated: 10^th^ December 2015). Participants were assured of the confidentiality and anonymity of the information they provided. No financial inducements were offered to participants.

### Data analysis

Data were entered into Statistical Process for Social Sciences (SPSS) software (Version 22.0 SPSS IBM, USA). Frequencies and percentages were used to report the respondents’ sociodemographic and knowledge characteristics. The chi-square test was used to investigate significant differences between categorical variables and the Mann-Whitney U test was used for ordinal variables. The limit for statistically significant differences was set at *p<*0.05.

## Results

### Demographic data

The questionnaire was completed by 476 undergraduate pharmacy students (8.3% response rate) from 14 universities in Australia and 466 undergraduate pharmacy students (63% response rate) from 6 universities in Sri Lanka. The majority of students [Australia (83%) and Sri Lanka (76%)] were aged between 20–25 years and were predominately female [Australia (73%) and Sri Lanka (67%)]. The demographic characteristics of the students are presented in Table 1.

**Table 1:** Characteristics of pharmacy undergraduates from Australian and Sri Lankan universities.

Characteristics	Australian students (n = 476)	Sri Lankan students (n = 466)	Significant level
	n (%)	n (%)	
Age:			
20–25	396 (83.2)	356 (76.4)	P<0.05
26–34	47 (9.8)	74 (15.9)	
35–44	24 (5)	26 (5.6)	
44+	9 (1.9)	10 (2.1)	
Gender:			
Male	127 (26.7)	151 (32.4%)	n.s.
Female	349 (73.3)	314 (67.4%)	
Level of study:			
1st year	109 (22.9)	139 (29.8)	P<0.05
2nd year	143 (30.7)	121 (25.9)	
3rd year	107 (22.5)	116 (24.9)	
4th year	117 (24.6)	90 (19.3)	

n. s: non significance

### Comparison of self-reported antibiotic use

The pattern of reported antibiotic use by pharmacy students from Australia and Sri Lanka are summarized in Table 2. Participants commonly reported previous antibiotic use [Australia (88%) and Sri Lanka (86%)]. The majority of students [Australia (89%) and Sri Lanka (77%)] reported they obtained antibiotics with a doctor’s prescription dispensed from a pharmacy [Australia (89%) and Sri Lanka (90%)]. More than half the students from both countries [Australia (63%) and Sri Lanka (57%)] reported receiving advice from a health care professional when obtaining the antibiotics. Purchasing antibiotics through market stalls/ hawkers was not reported as an option among the pharmacy students in either country [Australia (0.2%) and Sri Lanka (0.2%)]; nor was purchasing antibiotics through online stores [Australia (0.2%) and Sri Lanka (0%)]. A sizeable proportion of Sri Lankan students (19%) reported they obtained antibiotics without a prescription and presumably bought over the counter at pharmacies. Australian and Sri Lankan pharmacy students had mixed views about the optimal duration of antibiotic courses. The majority of the pharmacy students believed that the duration of antibiotic use should be completed as prescribed [Australia (95%) and Sri Lanka (72%)], while a proportion of pharmacy students reported that it is better to stop antibiotic therapy when a person feels better [Australia (4%) and Sri Lanka (21%)].

**Table 2:** Frequency and percentage of response for the questions related to antibiotic consumption from Australian and Sri Lankan students.

Questions	Australian students (n = 476)	Sri Lankan students (n = 466)
	n (%)	n (%)
*When did you last take antibiotics*?		
In the last month	40 (8)	132 (28)
In the last 6 months	98 (21)	170 (36)
In the last year	83 (17)	50 (11)
More than a year ago	199 (42)	51 (11)
Never	13 (3)	2 (0.4)
Can't remember	43 (9)	59 (13)
*Did you get the antibiotics from a doctor’s prescription*?		
Yes	422 (89)	359 (77)
No	31 (6)	87 (19)
Can't remember	23 (5)	12 (3)
*Did you get advice from a doctor*, *nurse or pharmacist on how to take them*?		
Yes	301 (63)	267 (57)
No	108 (23)	165 (35)
Can't remember	67 (14)	26 (6)
O*n that occasion*, *where did you get the antibiotics*?		
Pharmacy	425 (89)	420 (90)
Stall / hawker	1 (0.2)	1 (0.2)
The internet	1 (0.2)	0
Friend / family member	9 (2)	10 (2)
Saved from previous experience	4 (0.8)	10 (2)
Somewhere / Someone	13 (3)	4 (0.8)
Can't remember	23 (5)	9 (2)
*When do you think you should stop taking antibiotics once you’ve begun treatment*?		
When you feel better	19 (4)	99 (21)
When you have taken all	451 (95)	334 (72)
Don't know	6 (1)	30 (6)

### Comparison of knowledge about antibiotics

**Fig 1:**
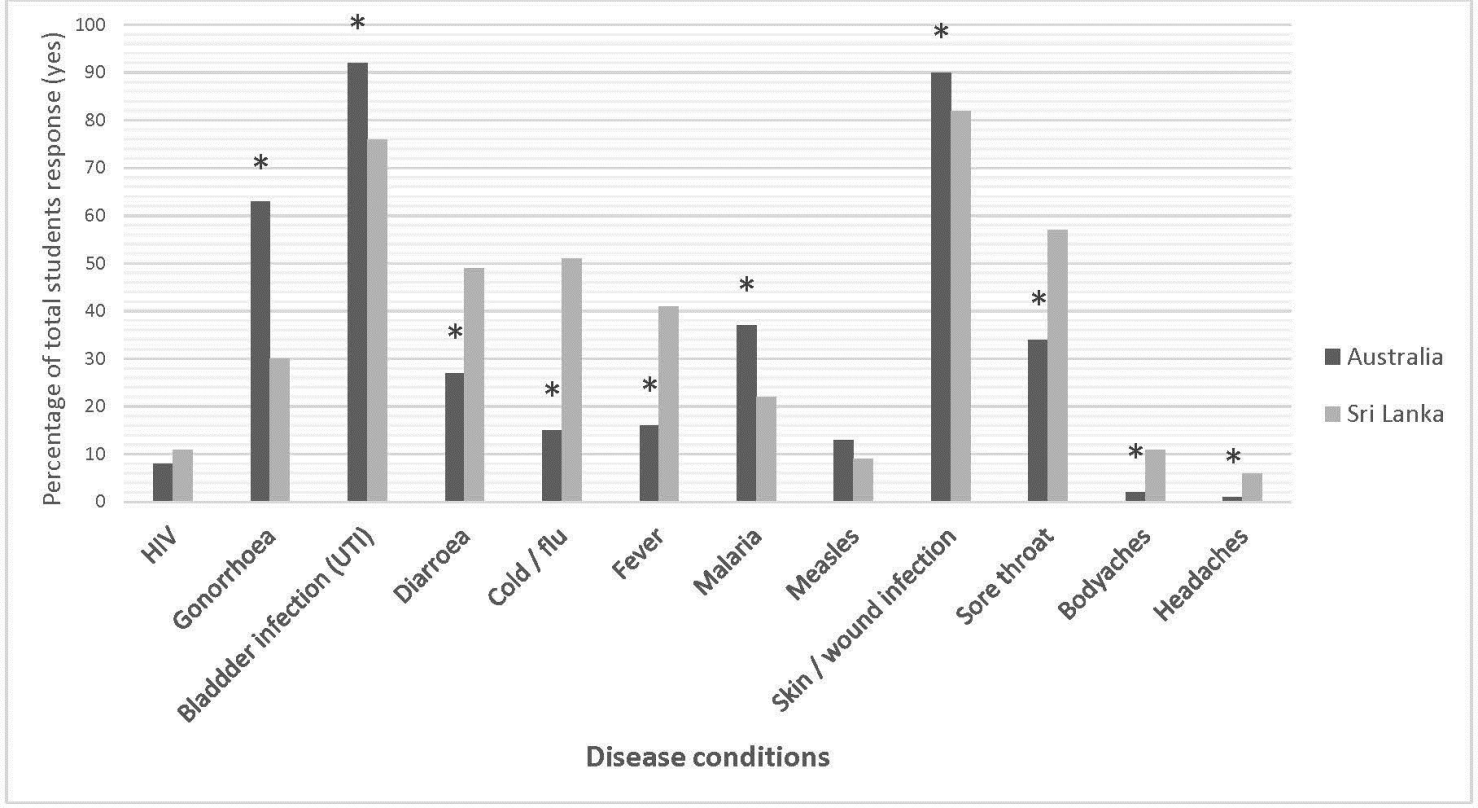
Percentage of total responses of pharmacy students from Australian and Sri Lankan universities reported antibiotics can be consumed for the above disease conditions. Asterisk denotes p<0.05, in comparison of Australian and Sri Lankan pharmacy students.

This study examined the students’ overall knowledge of antibiotics (Fig 1). Students were asked to indicate whether antibiotics were appropriate for certain health conditions. Australian pharmacy students displayed a greater overall knowledge regarding the appropriate antibiotic use for the different disease conditions when compared to Sri Lankan pharmacy students. A significantly greater number of Australian pharmacy students correctly reported that antibiotic use was appropriate for the management of gonorrhoea (63%), bladder infection (92%) and skin wound infection (90%) when compared to Sri Lankan students (30%, 76% and 80% respectively, *P*<0.05). Moreover, a significantly higher percentage of Sri Lankan pharmacy students incorrectly indicated that antibiotic use was appropriate for cold and flu symptoms (51%), body aches (11%), and headaches (6%) when compared to Australian students (15%, 2% and 1% respectively, p<0.05). Approximately half of the Sri Lankan pharmacy students reported antibiotics consumption is appropriate for sore throat (57%) and diarrhoea (49%) compared to Australian students (34%, and 27%, respectively, p<0.05).

### Comparison of knowledge about terminology related to antimicrobial resistance

The majority of participants reported they were familiar with the following terms related to antimicrobial resistance: antibiotic resistance [Australia (98%), Sri Lanka (94%) *p =* 0.014], antimicrobial resistance [Australia (88%), Sri Lanka (76%) *p<*0.0], drug resistance [Australia (92%), Sri Lanka (80%) *p<*0.0] and antibiotic resistant bacteria [Australia (94%), Sri Lanka (88%) *p =* 0.001]. However, students reported non-familiarity with the common acronym for ‘antimicrobial resistance’, AMR, [Australia (38%), Sri Lanka (12%) *p<*0.0]. Furthermore, Sri Lankan pharmacy students were unfamiliar with the terminology ‘superbugs’ [Australia (86%), Sri Lanka (10%) *p<*0.0].

### Comparison of knowledge of antibiotic resistance

Table 3 displays the results from the surveys questions that examined AMR knowledge. Overall, Australian pharmacy students showed a higher level of knowledge compared to Sri Lankan pharmacy students [median (IQR): 7 (7–8) vs. 5 (4–7) (*p*<0.001]. The majority of Australian pharmacy students (57%) correctly answered for the false statement “a*ntibiotic resistance occurs when your body becomes resistant to antibiotics and they no longer work as well”* compared to Sri Lankan pharmacy students (28%). The majority of students from both countries were aware of the spread of resistant bacteria and agreed [Australia (96%) and Sri Lanka (67%)] with the statement, *“many infections are becoming increasingly resistant to treatment by antibiotics”*.

**Table 3:** Knowledge of AMR reported by pharmacy students from Australia and Sri Lanka for the following statements related to AMR.

Statements	Australia (N = 476)^a^	Sri Lanka (N = 466) ^a^	P* value
	n (%)	n (%)	
Antibiotic resistance occurs when your body becomes resistant to	272 (57)	130 (28)	<0.001
antibiotics and they no longer works as well			
Many infections are becoming increasingly resistant to treatment by	459 (96)	313 (67)	<0.001
antibiotics			
If bacteria are resistant to antibiotics, it can be very difficult or	455 (96)	374 (80)	<0.001
impossible to treat the infections they cause			
Antibiotic resistance is an issue that could affect me or my family	461 (97)	335 (72)	<0.001
Antibiotic resistance is an issue in other countries but not here	458 (96)	384 (82)	<0.001
Antibiotic resistance is only a problem for people who take antibiotics	424 (89)	326 (70)	<0.001
regularly			
Bacteria which are resistant to antibiotics can be spread from person to person	415 (87)	276 (59)	<0.001
Antibiotic-resistant infections could make medical procedures like	452 (95)	304 (65)	<0.001
surgery, organ transplants and cancer treatment much more difficult			

^a^ Number (and %) of students correctly answering each question

* Statistical significance

### Comparison of knowledge about antibiotic use in the community

This study also examined students’ knowledge regarding the broader use of antibiotics in agriculture and in food-producing animals. The majority of Australian pharmacy students (60%) reported that they know about antibiotics usage in agriculture and food products compared to Sri Lankan pharmacy students (38%) *p<*0.00.

## Discussion

This study investigated self-reported antibiotic use, knowledge of antibiotics and AMR among undergraduate pharmacy students in Australia and Sri Lanka. Antibiotic use was highly prevalent among undergraduate pharmacy students in both countries. Greater knowledge of antibiotics and AMR were noted among Australian pharmacy students. Nationally accredited pharmacy curricula in Australia, well-monitored medicines regulations, availability of qualified pharmacists in community pharmacies and a higher overall socioeconomic status in Australia might be factors that have contributed to the reported appropriate antibiotic use among pharmacy students in Australian universities. In contrast, Sri Lankan undergraduate pharmacy students appeared to have some misconceptions about antibiotic use and a lower level of knowledge about antibiotics and AMR. This would suggest the need to improve pharmacy education and knowledge about antibiotics amongst pharmacy students in Sri Lanka.

It is notable that this study demonstrated highly prevalent antibiotic consumption in both countries. The majority of students purchased antibiotics with a prescription from a pharmacy. However, a significantly higher percentage of pharmacy students from Sri Lankan universities reported they obtained antibiotics without a prescription. Implementation and monitoring of national health policies and medicines regulations in both countries likely play a role in influencing access to antibiotics without a prescription. Antibiotic acquisition without prescription is a common practice [20] among university students in many developing countries: India [21], Pakistan [22], China [23], Palestine [24], Nigeria [25] and Brazil [26]. These studies have reported that consumption of antibiotics without appropriate prescription and pharmacist dispensing is associated with overuse and misuse of antibiotics, which in turn contributes to the development and spread of antibiotic resistance in developing countries. In contrast, very few pharmacy students from Australian universities reported antibiotic use without a prescription. This is probably due to the restricted access to antibiotics in Australia. They cannot be obtained over the counter from pharmacies or other stores but must be obtained by prescription. Furthermore, in Australia, a qualified pharmacist must always be available when a community pharmacy is open in order to dispense prescribed medicines and provide counselling for any dispensed medicine. The only other means to obtain an antibiotic in Australia is to access the unused portion of someone else’s antibiotics [27]. In contrast, in Sri Lanka, most pharmacies are managed by unqualified personnel [28]. Similar situations of unavailable qualified and trained pharmacists has been reported in the neighbouring countries of India [29] and Pakistan [30].

In this study, the majority of pharmacy students from Australia and Sri Lanka reported that antibiotic course should be completed according to the prescribed duration, while a few students from both countries reported that antibiotics should be stopped when the consumer feels better. The duration of antibiotic therapy is a contentious topic [31],[32]. Studies have emphasized that antibiotics should be stopped when the patient feels better [33]. However, in this study the majority of pharmacy students from both countries supported the concepts that an antibiotic course should be completed, or antibiotics should be taken as prescribed. Undergraduate pharmacy education in both countries may have influenced their belief that antibiotic course completion is preferred. Moreover, significant gaps are found between objectives and outcomes of modules taught in undergraduate pharmacy programs in Australian universities [34], [35].

This study found that pharmacy students from Australian universities demonstrated a higher level of knowledge about appropriate antibiotic use for certain disease conditions. The majority of the Australian pharmacy students correctly indicated antibiotic use was appropriate for gonorrhoea, bladder infection and skin infection. In contrast, the majority of the Sri Lankan pharmacy students incorrectly reported that antibiotic consumption was appropriate for cold and flu, body aches and headaches. This is an important research finding and highlights the risk of antibiotic misuse by Sri Lankan study participants. It follows that undergraduate pharmacy students in Sri Lankan universities have received inadequate education and training compared to pharmacy students in Australian universities. Further, the majority of Sri Lankan pharmacy students reported that antibiotics consumption is appropriate for sore throat and diarrhoea in contrast to Australian students. This suggests a potential for antibiotic overuse, as the majority of diarrhoea illnesses do not require antibiotic therapy [36]. Similarly illnesses involving a sore throat do not usually require antibiotic therapy as it is most commonly a self-limiting viral illness [37]. Non-judicious and inappropriate antibiotic choices can lead to the development of resistant bacteria [38]. Furthermore, these gaps in knowledge about antibiotics among future pharmacists of Sri Lanka provide a strong rationale for developing a new curriculum to enhance the knowledge regarding antibiotics and AMR. This approach aligns with the International Pharmaceutical Federation (FIP) Workforce Development Goals (WDG) [39] highlighting the need for a multidimensional approach to building workforce capacity in the area of AMR.

Pharmacy students from Australian universities tended to demonstrate greater knowledge than pharmacy students from Sri Lankan universities regarding the terminology related to AMR. An earlier report from Pakistan conducted among university students [22], highlighted the importance of knowing the terms related to AMR. In this study, the commonly used acronym, AMR was largely unknown by students in both countries. The terminology, superbugs, was unfamiliar to the majority of the Sri Lankan students. The reason for this is unclear and may relate to the wide use of the native languages (Sinhala and Tamil) with family and friends and in the media. There are probably very few opportunities to learn the terminology (and possibly the supporting principle) if students do not hear the term through their undergraduate study.

Pharmacy students from Australian universities showed greater knowledge towards AMR than Sri Lankan students when assessed through certain statements. Notably, it appears that the mechanisms that lead to the development of resistant bacteria is misunderstood by the majority of the Sri Lankan pharmacy students. This is critical knowledge for the practicing pharmacist given their role in antibiotic supply and associated counselling. A study conducted among Malaysian pharmacy students at public universities [40] showed a high level of understanding of antibiotic resistance. Importantly the pharmacy curricula in Malaysian universities cover aspects related AMR extensively. Given that Malaysia is considered a developing country, these findings have significant implications for the Sri Lankan undergraduate pharmacy curricula. Additionally, there is a need for enhanced clinical and ward-based training for Sri Lankan pharmacy students [6], [17] Clinically trained pharmacists can emerge themselves as role models in the multidisciplinary healthcare team to facilitate appropriate antibiotic use [41]. Some Sri Lankan universities have had difficulties in teaching clinical pharmacy modules and providing ward- based training due to access to academic staff with relevant expertise. This has led to international collaborations to teach clinical pharmacy to students and train local academics [6], [17]. This has the potential to influence the learning of pharmacy students in Sri Lanka and remains an ongoing challenges that is being addressed by initiatives such as CASPPER (Collaboration of Australians and Sri Lankans for Pharmacy Practice, Education and Research) [6], [14–17]

This study was conducted among undergraduate pharmacy students in two countries with different undergraduate pharmacy curricula, national policies and socioeconomic status. Further, this study was conducted as a nationwide survey in both countries, and data were collected from 14 universities in Australia and 6 universities in Sri Lanka. To our knowledge this is the first study to report a comparative analysis of antibiotic consumption and knowledge about antibiotics and AMR between Australia and Sri Lanka.

However, this study had some limitations. The online survey from Australian universities received a low response rate. In a study conducted among pharmacy students in 12 pharmacy schools in USA, Justo *et al*., [42] also reported a low response rate using an online survey on antimicrobial use. Another study conducted in South Africa using online participation, which assessed the knowledge and perceptions of antimicrobial stewardship concepts among final year pharmacy students in pharmacy schools across South Africa, also received a lower response rate [43]. Furthermore, this study used anonymous responses and voluntary participation, so students who were more academically engaged and interested in this topic might have been more likely to complete the online survey, potentially limiting the generalisation of the results. Lastly, some major themes related to appropriate antimicrobial use and AMR such as using antibiotics with a narrow spectrum and issues around oral vs *IV* antibiotic use have not been investigated in this study.

The results of this study support integrating modules on prudent antibiotic use and the factors related to AMR development into the undergraduate pharmacy curricula of Sri Lankan universities. One solution to address knowledge gaps regarding antibiotics could be the incorporation of AMR education as a requirement for individual pharmacy students’ revalidation and accreditation. It is also equally important to analyse the contents of Australian undergraduate pharmacy programme. Australia has a number of requirements to ensure an educational standard is achieved by all universities delivering courses for pharmacist qualification. These include mandated components in the university pharmacy curricula, an accreditation requirement for university courses every 5 years, and demonstration of professional competencies to be registered as a pharmacist, which is overseen by a national pharmacy council. These strategies should be considered by Sri Lankan academics and policy makers to raise the quality of pharmacy education and the performance of the pharmacy profession in Sri Lanka.

An initial start using relevant education and training should ensure future pharmacists will practice strategies that lead to appropriate antibiotic consumption and prudent dispensing practices. Postgraduate courses focusing on the management of infectious diseases and the development of antimicrobial resistance in Sri Lankan universities could be an additional strategy to improve antibiotic use. Furthermore, continuous professional education, such as workshops, seminars, and online courses on this subject area are another important means to improve appropriate antibiotic use and dispensing practices in Sri Lanka. These educational strategies would have a direct influence on the antibiotic dispensing practices of future pharmacists in Sri Lanka. In addition, using pharmacists to provide enhanced pharmaceutical care services in the hospital and community settings and pharmacist integration into the healthcare team has the potential to make a positive impact on the healthcare system in Sri Lanka.

Sri Lanka has introduced a national strategic plan for combating AMR [44] which adheres to the World Health Organisation (WHO) guidelines on AMR [3] for implementation between 2017 and 2022. This national plan emphasises the important role of health care professionals, educators, policymakers and other stakeholders including pharmacists to overcome AMR in Sri Lanka [44]. This research provides a way forward for Sri Lankan authorities and policy makers to take action on AMR by supporting strategies such as adequate education and training for pharmacists. This will minimize inappropriate antibiotic use and the growth of AMR, and in particular describes the role that improved education on AMR and the greater involvement of pharmacists can have in meeting the goals of this national plan.

Future research will involve identifying gaps in knowledge about appropriate antibiotic use in the various strata of undergraduate pharmacy students in these two countries. This important data needs to be feedback to the Sri Lankan government, health department, and universities. Furthermore, this data will help to identify the influence of each year of undergraduate study in Australian and Sri Lankan universities that has contributed to the knowledge of antibiotics and AMR. Additional research is needed to determine the optimal format for training and specific contents of the education. This will involve the implementation of a strategic educational plan for undergraduate pharmacy students in Sri Lankan universities.

## Conclusions

This study provides an understanding about antibiotic use and knowledge of AMR among pharmacy students in a developed country, Australia and a developing country, Sri Lanka. Antibiotic use was highly prevalent among undergraduate pharmacy students in both countries. These findings identify some misconceptions about antibiotics and a lower level of knowledge of AMR among Sri Lankan undergraduate pharmacy students. This has the potential to increase the inappropriate use of antibiotics in Sri Lanka. This research provides a way forward for Sri Lankan authorities and policy makers to take action on AMR by supporting strategies such as adequate education and training for pharmacists. This will minimize inappropriate antibiotic use and the growth of AMR, and in particular describes the role that improved education on AMR and the greater involvement of pharmacists can have in meeting the goals of this national strategic plan for combating AMR.

## Supporting information

S1 Table This is the S1 Table. Title: Characteristics of pharmacy undergraduates from Australian and Sri Lankan universities.
(XLSX)

S2 Table This is the S2 Table. Title: Frequency and percentage of response for the questions related to antibiotic use.
(XLSX)

S3 Table This is the S3 Table. Title: Response for the disease conditions and knowledge on antibiotics.
(XLSX)

S4 Table This is the S4 Table. Title: Response for the terminology related to AMR.
(XLSX)

S5 Table This is the S5 Table. Title: Number (and %) of students correctly answering each statement.
(XLSX)

S6 Table This is the S6 Table. Title: Percentage of students reported 'Yes' for question.
(XLSX)
